# The Development of a Novel Broad-Spectrum Influenza Polypeptide Vaccine Based on Multi-Epitope Tandem Sequences

**DOI:** 10.3390/vaccines13010081

**Published:** 2025-01-17

**Authors:** Song Zhao, Junhao Luo, Wenhui Guo, Li Li, Siyu Pu, Libo Dong, Wenfei Zhu, Rongbao Gao

**Affiliations:** 1 NHC Key Laboratory of Biosafety, National Institute for Viral Disease Control and Prevention, Chinese Center for Disease Control and Prevention, Beijing 102206, China; 13206880326@163.com (S.Z.); sgs442990378@163.com (J.L.); b2024007001@student.pumc.edu.cn (W.G.); lili@ivdc.chinacdc.cn (L.L.); wananpsy@163.com (S.P.); donglb@ivdc.chinacdc.cn (L.D.); zhuwf@ivdc.chinacdc.cn (W.Z.); 2 NHC Key Laboratory of Medical Virology and Viral Diseases, National Institute for Viral Disease Control and Prevention, Chinese Center for Disease Control and Prevention, Beijing 102206, China

**Keywords:** influenza, universal influenza vaccine, polypeptide vaccine, tandem epitopes, hemagglutinin, neuraminidase, Ii-Key

## Abstract

Background: Polypeptide vaccines have the potential to improve immune responses by targeting conserved and weakly immunogenic regions in antigens. This study aimed to identify and evaluate the efficacy of a novel influenza universal vaccine candidate consisting of multiple polypeptides derived from highly conserved regions of influenza virus proteins hemagglutinin (HA), neuraminidase (NA), and matrix protein 2 (M2). Methods: Immunoinformatics tools were used to screen conserved epitopes from different influenza virus subtypes (H1N1, H3N2, H5N1, H7N9, H9N2, and IBV). A polypeptide vaccine, P125-H, was constructed by linking multiple epitopes using Ii-Key technology. The immunogenicity of P125-H was assessed in mice using MF59-adjuvanted P125-H via intraperitoneal injection. Hemagglutination inhibition (HI) and neutralizing antibody responses were measured, along with IFN-γ levels in spleen lymphocytes. Protective efficacy was evaluated using viral challenge with lethal doses of H1N1 and H7N9. Results: Mice immunized with P125-H generated high levels of HI and neutralizing antibodies against multiple influenza strains. IFN-γ production was significantly elevated in spleen lymphocytes upon stimulation with the vaccine. P125-H protected mice from influenza infection, reducing weight loss and the viral load in the lungs, mitigating lung pathology, and decreasing mortality. Conclusions: The P125-H vaccine induced broad cross-protection against multiple influenza strains and elicited robust immune responses. It demonstrates strong potential as a candidate for a universal influenza vaccine.

## 1. Introduction

Influenza viruses are among the most prevalent pathogens causing respiratory infections in humans. Beyond their role in triggering occasional global pandemics, which can result in mortality figures ranging from hundreds of thousands to millions, influenza viruses also cause annual seasonal epidemics worldwide, leading to significant morbidity and substantial economic burden [1,2]. According to the World Health Organization, seasonal influenza leads to approximately 1 billion infections globally each year, with 3 to 5 million severe cases and 290,000 to 650,000 related deaths [3]. Currently, vaccination remains the most effective strategy for preventing influenza and mitigating its associated severe outcomes or mortality. However, the heterogeneity of influenza viruses, characterized by multiple types and subtypes, along with the frequent antigenic drift and reassortment between subtypes, poses significant challenges for existing vaccines [4]. These vaccines often lack cross-protective efficacy across different subtypes, resulting in reduced or even negligible protection when vaccine strains do not match circulating strains. Furthermore, the absence of cross-protection limits the utility of current vaccines in responding to emergent influenza pandemics. As a result, the development of a universal influenza vaccine remains a high priority in influenza vaccine research. Although no universal influenza vaccine has been approved to date, numerous studies suggest the feasibility and potential of such a vaccine [5,6,7].

An ideal universal vaccine would include epitopes that are highly conserved and immunogenic within the influenza virus, allowing it to overcome the challenges posed by antigenic drift and shift and providing robust, long-lasting cross-protection against various influenza virus subtypes [8]. Multi-epitope polypeptide vaccines are capable of presenting weakly immunogenic regions of entire antigenic molecules while forming intrinsic adjuvants through short peptide linkers, thereby enhancing the elicited immune response [9,10,11]. Through the tandem fusion of multiple antigenic epitopes, polypeptide vaccines could theoretically achieve cross-protection against a wide range of influenza virus subtypes, aligning with the objectives of universal vaccine development. Their small molecular size, ease of synthesis, and stability render them promising candidates for universal influenza vaccine development [12]. In terms of immunogen selection, HA, NA, and M2 are three highly promising targets for the design of universal influenza vaccines. HA and NA are the two most critical surface proteins of the influenza virus. HA existing as a trimer on the viral envelope facilitates viral entry by binding to host cell receptors, and the subunit HA2, which was considered as a focal target for universal influenza vaccine, has been shown to induce cross-reactive neutralizing antibodies against multiple influenza viruses [13,14]. NA is an essential enzyme in the release of new viral particles from infected cells. NA-induced specific antibodies can provide significant protection in severe influenza cases when the HA antigen of a vaccine strain does not match the circulating strain [15,16]. M2e, the extracellular domain of the M2 protein, consists of 23 amino acid residues that are highly conserved across various influenza virus subtypes. Although M2e-specific monoclonal antibodies do not exhibit significant hemagglutination inhibition activity, they can significantly reduce lung viral load in immunized mice following viral challenge [17]. Multiple M2e-based vaccines, including VLP vaccines, DNA vaccines, and synthetic peptide vaccines, are currently in development [18,19].

Furthermore, antigenic polypeptides need to be present on the surface of immune cells to activate immune responses by the major histocompatibility complex (MHC) class II molecules in antigen-presenting cells. Before binding antigenic peptides, these MHC II molecules are occupied by the invariant chain (Ii chain) in their antigen-binding grooves. The CLIP region and transmembrane domain of the Ii chain contain a key active motif (Ii-Key) that enhances antigen presentation [20]. In recent years, Ii-Key has emerged as a critical immunological carrier and modulator, capable of effectively aggregating with MHC class II molecules to activate specific T cells, stimulate IFN-γ secretion, and enhance both cellular and humoral immunity [21]. In this study, we identified highly conserved polypeptide sequences with strong immunogenic epitopes from the HA, NA, and M2 proteins of influenza viruses and linked them to the active peptide Ii-Key to construct a multi-epitope tandem influenza polypeptide vaccine aimed at broad-spectrum protection. We then evaluated its antigenicity, immunogenicity, and protective efficacy against influenza viruses in vitro or in animal models.

## 2. Materials and Methods

### 2.1. The Selection and Combination of Target Polypeptides for Components of a Potential Influenza Universal Vaccine

**Figure 1:**
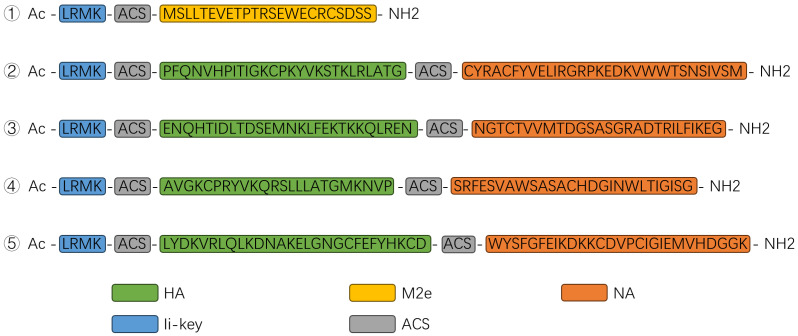
Formation of the five candidate polypeptides. LRMK denotes the Ii-Key sequence. Polypeptide ① is composed of the polypeptide of M2e and Ii-Key. Polypeptides ②–⑤ linked with the Ii-Key sequence represent the fusions of each polypeptide of HA and NA from different influenza viruses. ACS represents the linker, 6-aminohexanoic acid.

To identify high-quality linear epitopes from the highly conserved regions of the influenza virus HA, NA, and M2, we retrieved and downloaded DNA sequences of these segments, including those from WHO-recommended influenza vaccine strains (2020–2024) and various contemporary circulating subtypes from GeneBank. The software Mega5 was used to align these sequences and identify conserved polypeptide regions based on the DNA sequences. Then various prediction tools and algorithms were employed to evaluate the MHC-II binding affinity, CD4+ T-cell immunogenicity, protective antigen potential, or linear B-cell epitopes of these conserved polypeptides. Selection score setting for these prediction algorithms is shown in Table 1. Based on the combined performance metrics, a total of nine polypeptides were selected for further testing, including one from M2e, four from HA, and four from NA. Of those, the polypeptide from M2e linked with Ii-Key by a flexible linker (6-aminohexanoic acid, ACS) was set as a component of the designed vaccine, while the other four fused polypeptides with Ii-Key were formatted by each of the HA and NA polypeptides by ACS (Figure 1). These polypeptides were synthesized by Nanjing Synpeptide Biotechnology Co., Ltd., using solid-phase synthesis, with a purity exceeding 95%.

### 2.2. Antigenicity Evaluation of the Candidate Polypeptides

The polypeptide antigenicity was evaluated using an indirect ELISA to determine the binding affinity of the individual polypeptide to specific IgG antibodies against influenza viruses in mouse sera, or the specific IgG antibodies induced by themselves in the sera of immunized mice with those polypeptides. The HI antibody positive sera were used from survivals of mice infected with the H1N1, H5N1, or H7N9 influenza virus in our previous study. The HI antibody titers for these sera were 1:320, 1:320, or 1:1280 against H1N1, H5N1, or H7N9, respectively. In brief, a 96-well ELISA plate was coated with an individual polypeptide of the five candidates at a concentration of 5 μg/mL in 100 μL per well, followed by incubation at 4 °C overnight. After blocking with 1% BSA at 37 °C for 1 h, 100 μL of diluted sera (1:100) were added to the polypeptide-coated wells. The plate was incubated at 37 °C for 2 h. Following five washes with 1× PBST, HRP-conjugated secondary antibody (GAM007, Multisciences, Hangzhou, China) was added and incubated at 37 °C for 1 h. After five additional washes with 1× PBST, the TMB substrate (IT0001, LEAGENE, Beijing, China) was added and incubated at room temperature for 20 min. The reaction was stopped with the ELISA stop solution (C1058, Solarbio, Beijing, China), and the optical density (OD) was measured at 450 nm using an ELISA reader (Thermo Fisher Scientific, Waltham, MA, USA). The antigenicity of the individual polypeptide or polypeptide combinations was assessed based on the differences in OD values.

### 2.3. Immunogenicity Evaluation of the Candidate Polypeptides

To evaluate the immunogenicity of the candidate polypeptides, the polypeptides were inoculated in 6- to 8-week-old female SPF-grade C57BL/6J mice, which were obtained from Beijing Vital River Laboratory Animal Technology Co. Ltd. and housed at the Animal Center of the Chinese Center for Disease Control and Prevention (CDC). The animal experiments were approved by the Animal Ethics Committee of the Virus Disease Prevention and Control Institute, Chinese CDC (Ethical approval number: 20221107112, 20 January 2022). In brief, lyophilized polypeptides were reconstituted in 1 mL of DMSO. For immunization, polypeptide solutions were diluted to the working concentration in PBS. The polypeptide solutions were mixed with MF59 adjuvant at a 1:1 ratio, thoroughly homogenized using a syringe until a uniform emulsion was achieved. Mice were immunized with 200 μL of either polypeptide–adjuvant mixture or PBS-adjuvant mixture via intraperitoneal injection. Booster injections were administered at the same dosage on days 14 and 28 post-primary immunization. Three polypeptide combinations of ① + ② + ③, ① + ② + ④, or ① + ② + ⑤ were tested. Each combination was evaluated at a low dose (2.5 μg/polypeptide) and a high dose (10 μg/polypeptide), prospectively. An adjuvant-only control group was set with the immunization of PBS mixed with MF59. Blood samples were collected from the retro-orbital vein prior to each immunization for subsequent antibody assays. Fourteen days following the third immunization, spleens were harvested for the analysis of cellular immune responses. The polypeptide immunogenicity was determined by the levels of specific HI or neutralizing antibodies against influenza viruses in the sera of immunized mice, or IFN-γ levels in the spleen lymphocytes of the mice under the stimulation of the combined polypeptides.

### 2.4. Viral Challenge

To evaluate the protective efficacy of the immunization with the polypeptide combinations, the immunized mice were subjected to a viral challenge with A/California/04/2009 (H1N1) (H1N1-CA04) or the mouse-adapted H7N9 vaccine strain (PR8 backbone with HA and NA from A/Anhui/1/2013(H7N9), H7N9-MA). After deep anesthesia with isoflurane, the mice were intranasally inoculated with 50 μL of 1 × 10^5^ TCID50 (100-fold 50% lethal dose) H1N1-CA04, 2 × 10^2^ TCID50 (40-fold 50% lethal dose) H7N9-MA, or PBS. Weight changes and mortality rates were recorded over a 14-day period. On days 3, 7, and 14 post-infection, five mice from each group were euthanized, and serum and lung tissues were collected for antibody titer, viral load, lung-to-body weight ratio, or histopathological examination. Serum samples were stored at −20 °C. The left lung was washed with PBS and stored at −80 °C, while the right lung was fixed in 10% formalin and stored at room temperature. If the mice lost over 25% of their initial body weight, they were humanely euthanized and necropsied.

### 2.5. Measurement of Specific Anti-Influenza Virus Hemagglutination Inhibition Antibodies

Hemagglutination inhibition (HI) assays were used to determine the levels of anti-influenza virus antibodies in mouse sera. Mouse sera were mixed with receptor-destroying enzyme (RDE, Japan Bio Serum, 340016) at a 1:3 ratio and incubated at 37 °C for 16–18 h, followed by heat inactivation at 56 °C for 30 min. The inactivated sera were adsorbed with concentrated turkey red blood cells for 1 h to remove any nonspecific factors that might affect agglutination. The treated serum samples were diluted in PBS starting at a 1:10 dilution, and subsequently subjected to two-fold serial dilutions. The virus at a concentration of 4 HAU/25 μL was added, and the mixture was incubated at 37 °C for 1 h. Afterward, 50 μL of 1% turkey red blood cells was added to each well and incubated at room temperature for 30 min. The highest serum dilution that inhibited hemagglutination was considered the HI antibody titer of the serum.

### 2.6. Measurement of Specific Neutralizing Antibodies Against Influenza Virus

The specific neutralizing antibody levels against influenza viruses were determined using a micro-neutralization assay. MDCK cells used in this study were cultured and maintained at 37 °C in a 5% CO_2_ atmosphere in DMEM supplemented with 10% fetal bovine serum (FBS), 1% penicillin–streptomycin solution, and 2.5% HEPES. In brief, MDCK cells were seeded at 3 × 10^4^ cells per well in 100 μL of medium in a 96-well microtiter plate and incubated at 37 °C for 18–24 h. Subsequently, sera treated with RDE were serially diluted in PBS buffer and mixed with 100 TCID50 of H1N1-CA04, A/Beijing Xuanwu/241/2006 (H3N2), A/Hubei/1/10 (H5N1-RG), H7N9-MA, A/Hunanlengshuitan/11197/2013 (H9N2), or B/Guangdongluohu/151/2007 viruses in 50 μL volumes and incubated at 35 °C for 1 h. All of the above viruses were preserved at the National Influenza Center of China and were propagated in MDCK cells. The virus–serum mixtures (100 μL per well) were then transferred to the MDCK cell plates and incubated at 35 °C for 1 h. Following incubation, the wells were washed with PBS buffer, and DMEM containing 2 mg/mL TPCK-treated trypsin was added to the cells, which were then incubated at 35 °C for 72 h. Finally, 50 μL of supernatant from each well and 50 μL of 1% turkey red blood cells were added to 96-well V-bottomed hemagglutination plates and thoroughly mixed. After 30 min, the results were observed, with the absence of hemagglutination being considered positive for neutralization.

### 2.7. Assessment of Lymphocyte Immune Response Levels Induced by Combined Polypeptides in Immunized Mice

Lymphocyte immune response levels induced by combined polypeptides in immunized mice were evaluated using a Mouse IFN-γ ELISpot kit (551083, BD Biosciences, Franklin Lakes, NJ, USA). Fourteen days after the final immunization, spleens were harvested from each group of mice and mechanically dissociated to obtain single-cell suspensions. A total of 4 × 10^4^ spleen cells in 100 μL were plated per well in triplicate on a 96-well PVDF plate pre-coated with IFN-γ capture antibodies (5 μg/mL). The cells were then stimulated with the combined polypeptides at a low dose of 4 μg/polypeptide and a high dose of 12 μg/polypeptide, respectively, and incubated at 37 °C in a 5% CO_2_ environment for 24 h. After the cell–polypeptide mixtures were removed, the wells were washed thoroughly with wash buffer. Detection antibodies were then added, and the plates were incubated at room temperature for 2 h. Following additional washes, Streptavidin-HRP conjugate was added and incubated for 1 h at room temperature. After the final washing steps, the substrate solution (551951, AEC Substrate Reagent Set, BD Biosciences, San Jose, CA, USA) was added to develop the spots, which were observed for 5–60 min. The reaction was stopped with deionized water, and the plates were allowed to dry completely. The frequency of IFN-γ-producing lymphocytes was then quantified using an ELISpot analyzer (AID vSpot Spectrum, AID, Strassberg, Germany).

### 2.8. Virus Titration

The viral titers of all the viruses or lung tissues from mice were determined using the 50% tissue culture infectious dose (TCID50) method. The left lung of each mouse was homogenized thoroughly using a tissue grinder (9001272, Qiagen, Hilden, Germany) in 1 mL of PBS containing 2% penicillin–streptomycin and 5 mm diameter steel beads. Those homogenates were centrifuged at 3000 rpm for 15 min at 4 °C, and the supernatants were collected for TCID50 titration. Briefly, 3 × 10^4^ MDCK cells in 100 μL were seeded into each well of a 96-well microtiter plate (3603, Corning, New York, USA) and incubated at 37 °C for 18–24 h. Serial dilutions of each virus or supernatant sample were prepared in DMEM medium containing 1% bovine serum albumin and 2 μg/mL TPCK-treated trypsin (T819144, FEIMOBIO, Shanghai, China). Each diluted virus or sample in 100 μL was added to the MDCK cells, with four replicates per sample. After 72 h of incubation at 35 °C, 50 μL of the supernatant from each well was transferred to a 96-well V-bottom plate (B-PYB96JW, BKMAM, Changde, China), followed by the addition of 50 μL of 1% turkey red blood cells (TRBC) and thorough mixing. After 30 min of incubation at room temperature, wells showing hemagglutination were considered positive. The TCID50 was calculated using the Reed–Muench method based on the ratio of positive to negative wells.

### 2.9. Quantification of IFN-γ in Mouse Serum

The levels of IFN-γ in mouse serum on days 3 and 7 post-infection were quantified using the Quantikine™ ELISA Mouse IFN-γ Immunoassay kit (MIF00, R&D Systems, Minneapolis, MN, USA), according to the manufacturer’s instructions. The optical density (OD) at 450 nm was measured using a microplate reader, and the IFN-γ concentration was determined using a standard curve.

### 2.10. Histopathological Evaluation and Immunohistochemical Staining

The right lung tissue of each mouse was fixed in formalin, followed by dehydration, clearing, and paraffin embedding using an automatic tissue processor (TP1020, Leica Biosystems, Nussloch, Germany) or a paraffin-embedding station (EG1150H, Leica Biosystems, Nussloch, Germany). Embedded lung tissues were sectioned into 4 μm thick slices using a microtome (RM2235, Leica Biosystems, Nussloch, Germany) for histopathological evaluation and immunohistochemical (IHC) staining. Histopathological evaluation was performed using a routine hematoxylin and eosin (H&E) staining. IHC staining was conducted to detect the distribution of influenza virus NP in lung tissues using an anti-influenza virus NP antibody (ab20841, Abcam, Cambridge, UK) and a two-step HRP detection kit (PV-9003-18mL, ZSGB-BIO, Beijing, China). The stained cells were observed under a microscope (Zeiss Axio Imager M2, Carl Zeiss AG, Oberkochen, Germany) to assess the staining patterns.

### 2.11. Statistical Analysis

All experimental data were plotted and statistically analyzed using GraphPad Prism software (Version 5.0). For quantitative data, an unpaired t-test was used for comparisons between two groups, while a one-way ANOVA was used for comparisons among three or more groups. Statistical significance was determined by a two-tailed test with a threshold of *p* ≤ 0.05. Data are presented as the mean ± standard error of the mean (SEM).

## 3. Results

### 3.1. Epitopes Analysis and Structural Characteristics of the Candidate Polypeptides

**Figure 2:**
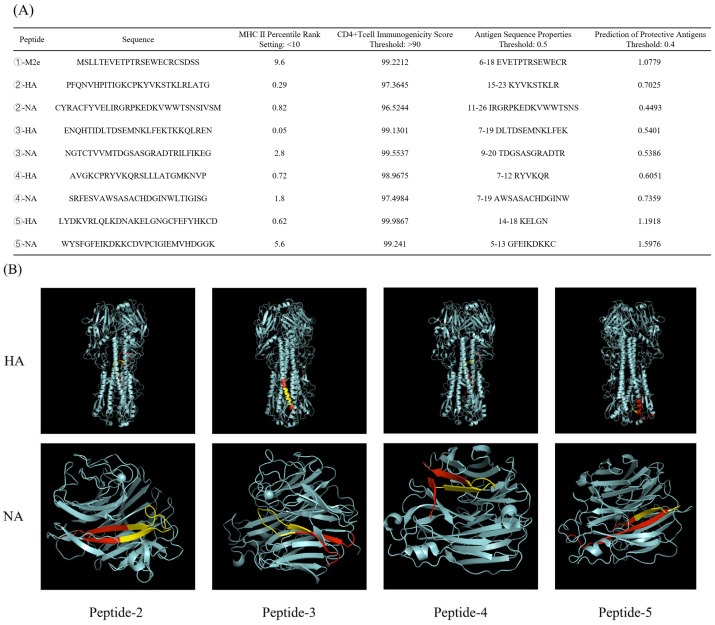
The scores of the candidate polypeptides in different prediction models and their positions in the complete protein. (**A**) The scores of the candidate polypeptides in MHC class II molecule binding ability, CD4+ T-cell immunogenicity, and linear B-cell epitope prediction of protective antigens. (**B**) The spatial positioning of polypeptides ②–④ within the complete HA or NA protein structures was visualized using PyMOL 3.0, with these regions represented in red. Predicted linear epitopes within those polypeptide sequences were highlighted in yellow.

We conducted an extensive analysis on the selected polypeptides using multiple predictive models. The findings reveal that all selected polypeptides exhibited percentile ranks below 10% for binding affinity to MHC class II molecules and immunogenicity scores for CD4+ T cells exceeding 90% (Figure 2A). For the screening of combinations and enhancing the collaboration of NA and HA on antibodies response, a NA peptide was linked to each HA peptide. Peptides 2 to 4 correspond to the concatenated fragments of HA and NA, as shown in Figure 1. The three-dimensional structures (rendered using PyMOL 3.0) illustrated the specific locations of the HA and NA polypeptide fragments within the complete protein structures, highlighted in red and yellow. Notably, all selected polypeptide fragments contained at least one linear epitope (Figure 2B).

### 3.2. Antigenicity and Immunogenicity of Candidate Polypeptides and Cellular Immune Responses Induced by the Candidate Polypeptides

**Figure 3:**
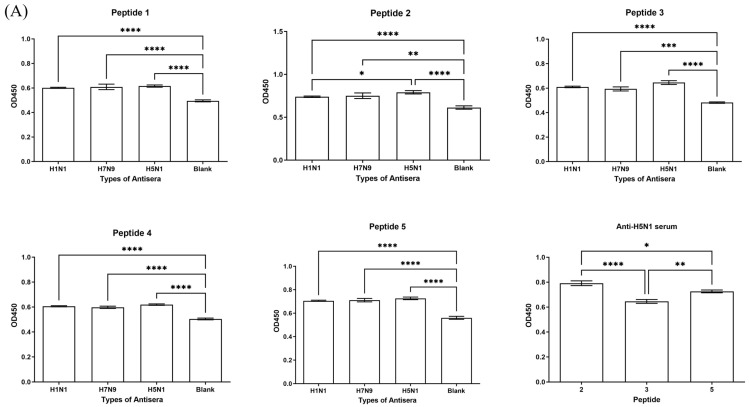

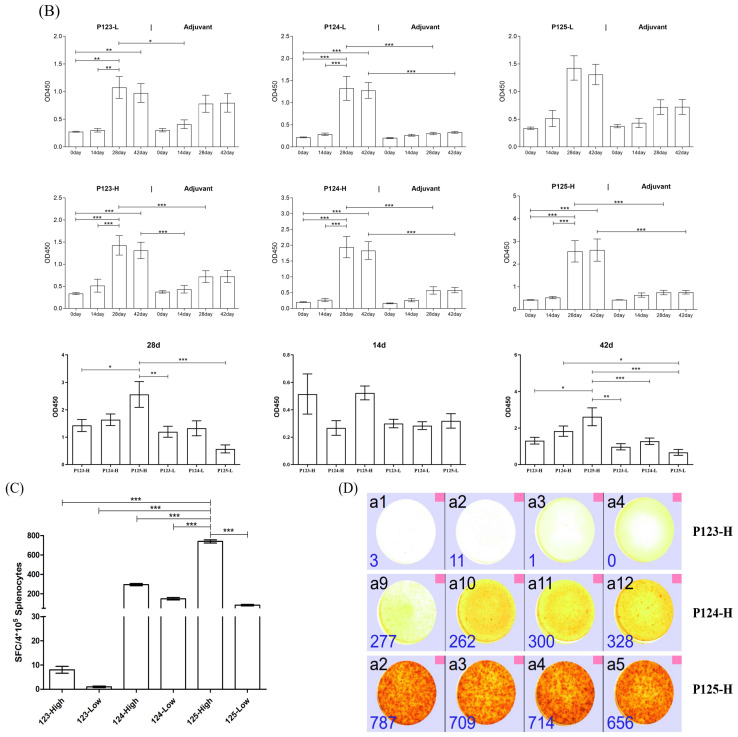
Antigenicity, immunogenicity, and induction of cellular immune responses by candidate polypeptides. (**A**) The binding affinity of five individual polypeptides to IgG antibodies in convalescent sera from mice infected with H1N1, H5N1, and H7N9 influenza viruses. The OD values at 450 nm represented the binding affinity levels. The control sera were from mice unexposed to any influenza virus (serum dilution, 1:100). (**B**) The IgG levels of specific antibodies in the sera of mice immunized with six different combinations of polypeptides, each targeting the polypeptide itself (serum dilution, 1:100). (**C**) The levels of IFN-γ production in mouse spleen lymphocytes stimulated with different polypeptide combinations (low dose: 4 µg/polypeptide; high dose: 12 µg/polypeptide), assessed by quantifying the number of spot-forming cells (SFCs) in the ELISpot assay. Statistical significance is denoted as follows: * *p* < 0.05, ** *p* < 0.01, *** *p* < 0.001, **** *p* < 0.0001 (two-tailed test). (**D**) The raw data of IFN-γ SFCs in mouse spleen cells stimulated with the P123(a1–a4), P124(a9–a12), and P125(a2–a5) polypeptide combinations at a dose of 12 µg/polypeptide. The number in the lower-left corner represents the count of SFCs per well.

The antigenicity or immunogenicity of the candidate polypeptides was evaluated by the levels of IgG antibodies in convalescent sera from influenza virus-infected mice or sera from the polypeptide-immunized mice using the polypeptide-coated ELISA. The results demonstrate that all five polypeptides exhibited varying degrees of specific binding to IgG in the sera of mice infected with H1N1, H5N1, and H7N9 influenza viruses. Compared to normal mouse sera, polypeptides ②, ③, and ⑤ showed significantly higher OD values of responses to IgG in sera from H5N1-infected mice, indicating a significantly stronger binding capacity to the convalescent sera of H5N1-infected mice. In comparison, the detected OD values of polypeptide ② were remarkably higher than those of polypeptides ③ and ⑤ (Figure 3A). Hence, to increase the broad spectrum of antibodies induced by polypeptides, we used three different combinations of polypeptides for the following immunization in mice: polypeptides ① and ② combined with polypeptides ③, ④, or ⑤, named P123, P124, or P125, respectively. The results show that different levels of specific IgG antibodies against the combined polypeptides were induced in mice. In comparison, the concentration of the specific antibodies were significantly increased after the second immunization, while there was no significant difference between antibody levels after the second immunization or the third immunization. In addition, P125 at a high dose (P125-H) induced much higher antibody levels after two immunizations than P125 at a low dose (P125-L), and P-123 or P-124 at low or high dose (Figure 3B), respectively.

To evaluate the cellular immune responses elicited by the combined polypeptides, splenic lymphocytes were harvested from immunized mice and subjected to ELISpot assays with the stimulation of combined polypeptides. The results reveal that all combined polypeptides effectively induced IFN-γ secretion in mouse splenic lymphocytes. In comparison, P125-H induced the highest levels IFN-γ with 740.2 ± 15.8 SFCs per 4 × 10^5^ splenocytes, while P123-L or P123-H induced less than 10 SFCs per 4 × 10^5^ splenocytes, and P124-H induced 294.4 ± 11.8 SFCs per 4 × 10^5^ splenocytes (Figure 3C,D).

### 3.3. The Levels of Antibodies Against Influenza Viruses Induced by the Candidate Polypeptides

The levels of antibodies against influenza viruses induced by the candidate polypeptides were tested by the HI assay and/or micro-neutralization assay. The results demonstrate that the mice immunized with each of the three polypeptide combinations produced varying levels of HI antibodies against H1N1-CA04, H7N9-MA, H3N2, and H5N1.

**Figure 4:**
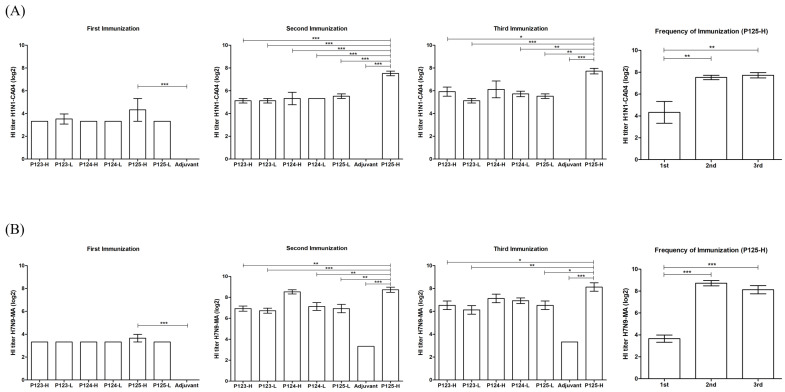

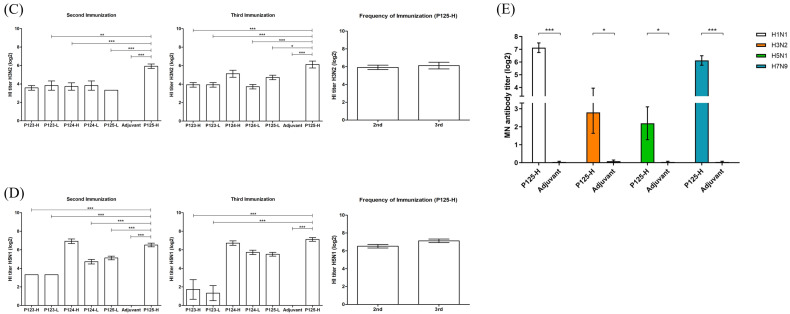
HI and neutralizing antibody titers against various influenza virus subtypes in mice sera following immunization with different polypeptide combinations. (**A**) HI antibody titers against H1N1-CA04 following immunization with three different polypeptide combinations. (**B**) HI antibody titers against H7M9-MA following immunization with three different polypeptide combinations. (**C**) HI antibody titers against H3N2 following immunization with three different polypeptide combinations. (**D**) HI antibody titers against H5N1 following immunization with three different polypeptide combinations. (**E**) Neutralizing antibody titers against various influenza virus subtypes following two immunizations with P125-H. Statistical significance was assessed using a one-way ANOVA, with *p*-values denoted as follows: * *p* < 0.05, ** *p* < 0.01, *** *p* < 0.001 (two-tailed).

The levels of HI antibodies against H1N1-CA04 or H7N9-MA in the sera of mice were not significantly increased compared to MOCK on the first immunization with the candidate polypeptides, except P125-H, but did significantly increase on the second and third immunizations. In comparison, the HI antibodies levels induced by P125-H were remarkable higher than those by P123, P124, and P125-L. The geometric mean titers (GMTs) of the HI antibodies were 20, 184, and 211 against H1N1-CA04, and 13, 422, and 278 against H7N9-MA after the three immunizations with P125-H, while there was no significant difference between the levels of the second immunization and those of the third immunization (Figure 4A,B). To follow the HI assay of H3N2 or H5N1, we only test the levels of antibodies in the sera of mice on the second and third immunizations due to insufficient serum samples collected on the first immunization. The results show that the antibody levels against either H3N2 or H5N1 were much higher in the sera of P125-H-immunized mice than those in P123-, P124-, or P125-L-immunized mice on either the second immunization or the third immunization, and there were no significant differences between the levels of the second immunization and those of the third immunization, although the antibody levels were slightly increased on the third immunization. The GMTs of antibodies were 61 and 70 against H3N2, and 93 and 139 against H7N9-MA after the two immunizations with P125-H (Figure 4C,D). However, no HI antibodies were detected against H9N2 or influenza B viruses in all groups.

In addition, the microneutralization assay showed that P125-H induced the production of neutralizing antibodies against H1N1-CA04, H7N9-MA, H3N2, and H5N1 on the second immunization, with GMTs of 139, 70, 7, and 4.6, respectively (Figure 4E). No neutralizing antibodies were detected against H9N2 or influenza B viruses.

### 3.4. Protective Efficacy of P125-H Immunization Against Different Influenza Virus Subtypes

**Figure 5:**
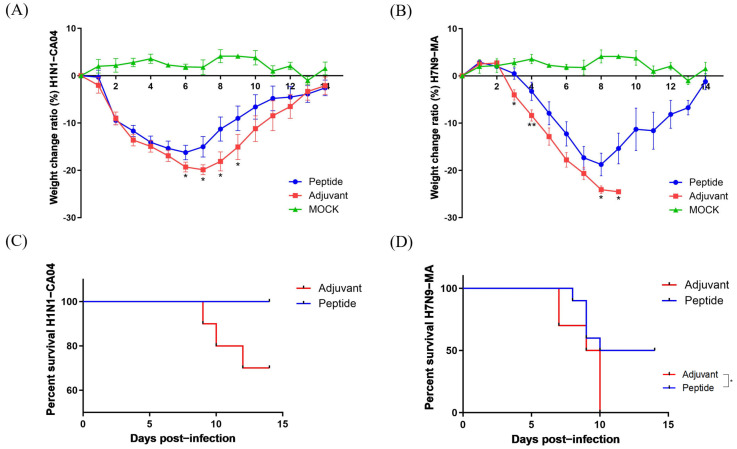
Weight changes and survival rates of P125-H or adjuvant-inoculated mice post-infection with H1N1-CA04 or H7N9-MA. (**A**,**B**) Recordings of weight loss in all surviving mice from the P125-H and adjuvant control groups over a 14-day period. (**C**,**D**) Kaplan–Meier survival curves are presented, with *n* = 10 per group. Statistical analyses were performed using a one-way ANOVA or log-rank test, with * *p* < 0.05 and ** *p* < 0.01 (two-tailed test).

To assess the protective efficacy of P125-H, mice were immunized with two doses of the polypeptides at 30 μg each, followed by infection with either H1N1-CA04 or H7N9-MA influenza virus. The data demonstrated that P125-H significantly alleviated the weight loss of mice with H1N1-CA04 or H7N9-MA infections compared to the adjuvant control group. The P125-H-immunized mice presented a peak weight loss of 16.23% and 18.75% on days 6 and 8 after H1N1-CA04 and H7N9-MA infection, respectively. In contrast, the adjuvant-inoculated mice, respectively, presented a peak weight loss of 19.9% and over 25% on days 7 and 9 after H1N1-CA04 and H7N9-MA infection (Figure 5A,B). The survival ratio also suggested that P125-H significantly increased the survival rates of mice infected with H1N1-CA04 or H7N9-MA. All (100%, *n* = 10) were survived, while seven (70%, *n* = 10) adjuvant-inoculated mice survived on day 14 after H1N1-CA04 infection. On day 14 after H7N9-MA infection, 50% survival rate (*n* = 10) was observed in P125-H-immunized mice, with an initial death on day 8 after infection, while 0% (*n* = 10) survival rate was observed in adjuvant-inoculated mice, with an initial death on day 7 (Figure 5C,D).

### 3.5. Effects of P125-H Immunization on Viral Load in Lung Tissue and Serum IFN-γ Levels in Infected Mice

**Figure 6:**
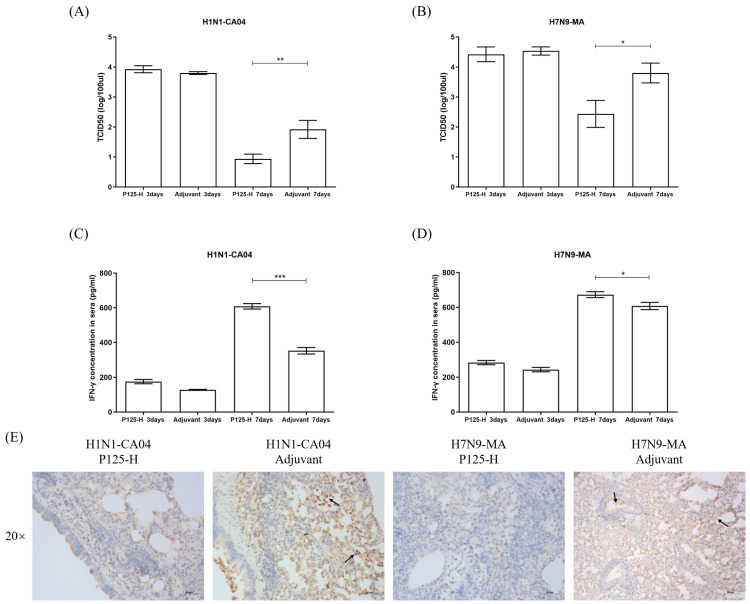
Viral load in lung tissue, distribution of influenza virus NP protein, and serum IFN-γ levels in mice. (**A**) TCID50 levels in the lung tissues of P125-H-immunized or adjuvant control mice infected with H1N1-CA04 on days 3 or 7 post-infection. (**B**) TCID50 levels in the lung tissues of P125-H-immunized or adjuvant control mice infected with H7N9-MA on days 3 or 7 post-infection. (**C**) IFN-γ levels in the sera of mice on days 3 or 7 after H1N1-CA04 infection. (**D**) IFN-γ levels in the sera of mice on days 3 and 7 after H7N9-MA infection. * *p* < 0.05, ** *p* < 0.01, and *** *p* < 0.001 (two-tailed test). (**E**) IHC stains for the NP protein of the influenza virus in lung tissues on day 7 after infection with H1N1-CA04 or H7N9-MA, with positive staining indicated by brown coloration (as shown by arrows). The original magnification used was eyepiece (10×) and objective (20×).

The TCID50 titration results reveal that P125-H immunization significantly reduced the viral load in the lung tissues of mice on day 7 after either H1N1-CA04 or H7N9-MA infection, although no significant difference shown on day 3 post-infection (Figure 6A,B). The IHC results also suggest that the virus distribution was more localized in the lung tissues of the immunized group than that in the non-immunized group on day 7 after infection with H1N1-CA04 or H7N9-MA. The viral stains were mainly distributed in bronchial epithelial cells in the lung tissues of immunized mice while being scattered in both alveolar and bronchial epithelial cells in the lung tissues of non-immunized mice on day 7 after infection with H1N1-CA04, whereas the virus was diffusely distributed in the bronchial and alveolar cells in the lung tissues of both immunized and non-immunized mice on day 7 after infection with H7N9-MA. In comparison, the density of the diffusely distributed virus in the lung tissues of the immunized group was significantly reduced on day 7 after infection with H7N9-MA (Figure 6E). The ELISA analysis demonstrated that, on day 7 post-infection with either H1N1-CA04 or H7N9-MA, the serum levels of IFN-γ in P125-H-immunized mice were markedly elevated compared to those in the adjuvant control group, indicating that the immunization with P125-H increased the innate immune response of the host after influenza infection (Figure 6C,D).

### 3.6. Effects of P125-H Immunization on Pulmonary Lesions in Infected Mice

**Figure 7:**
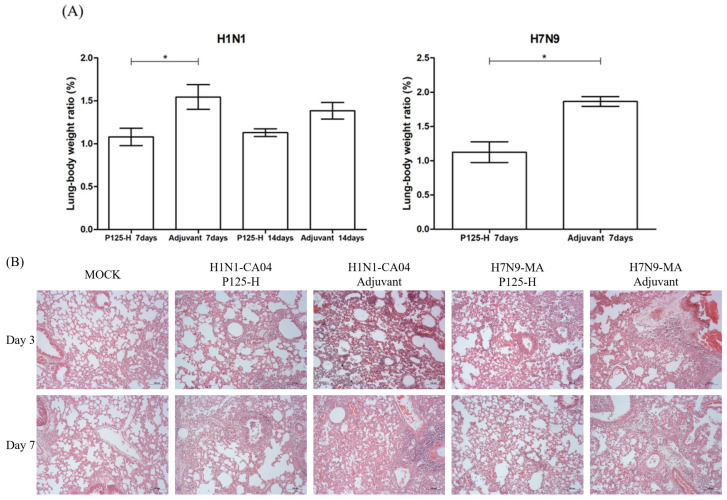
Lung-to-body weight ratio and pulmonary histopathological alterations in P125-H-immunized mice compared to adjuvant controls following H1N1-CA04 or H7N9-MA infection. (**A**) Lung-to-body weight ratios (LBs) in P125-H-immunized and adjuvant control mice at days 7 and 14 post-infection with H1N1-CA04 or H7N9-MA (*n* = 5). (**B**) Representative histopathological alterations in lung tissues at days 3 and 7 post-infection, visualized by hematoxylin and eosin (H&E) staining. Original magnification: eyepiece (10×) and objective (10×). Scale bar: 100 μm. H&E, hematoxylin and eosin. Statistical significance is denoted as follows: * *p* < 0.05 (two-tailed test).

The lung-to-body weight ratio (LB) analysis demonstrated that P125-H immunization reduced LB significantly on day 7 post-infection in mice challenged with H1N1-CA04 or H7N9-MA, compared to the adjuvant control group. And the LB still was reduced with a *p*-value approaching 0.05 in the P125-H group on day 14 post-infection with H1N1-CA04, although the difference was not statistically significant (Figure 7A). HE staining demonstrated that the lung tissues of mice presented a range of pathological damage, including bronchial epithelial cell desquamation, infiltration of inflammatory cells, pulmonary interstitial edema, and/or hemorrhage on days 3 and 7 post-infection with H1N1-CA04 and H7N9-MA, respectively. Compared to the adjuvant group, the immunized mice presented significantly reduced pathological damage in the lung tissues on day 7 post-CA04 infection, or on both days 3 and day 7 post-H7N9-MA infection, characterized by more localized pathological changes, decreased infiltration of inflammatory cells, and/or reduced pulmonary edema (Figure 7B).

## 4. Discussion

Generally, the degree of match between the vaccine strain and the circulating strain is a critical determinant of vaccine efficacy. And selecting the optimal vaccine strain is both a complex and time-sensitive process, typically requiring decisions to be made 6 to 8 months prior to vaccine distribution. This challenge stems from the need to predict which viral strains will be predominant during the upcoming influenza season, necessitating an early and precise determination based on global surveillance and epidemiological data [22]. The development of system dynamics-based predictive models utilizing global surveillance data holds the potential to accurately forecast future dominant viral strains. This approach could significantly improve the precision of vaccine strain selection, thereby enhancing overall vaccine efficacy [23]. Given the continuous challenge posed by antigenic drift and shift in influenza viruses, the development of a universal vaccine that provides lasting and broad protection remains a significant scientific endeavor [5]. The selection of immunogens is critical and marvelous in the design of such vaccines although the advancement of immunoinformatics offers powerful tools for identifying potential candidates [24]. AI-driven antigen design may be as a promising approach for generating novel, broadly reactive immunogens [25,26,27].

Currently, several polypeptide vaccines, including those targeting influenza viruses like Flu-v and Multimeric-001, are undergoing clinical trials, highlighting their potential in vaccine development [28,29]. However, polypeptide vaccines still possess inherent limitations, such as suboptimal immunogenicity and poor stability, therefore a strategic approach was employed in the design of our candidate polypeptides [30]. In this study, to increase the coverage of protective epitopes across influenza viruses, we focused on polypeptides derived from three different proteins of HA, NA, and M2, which are surface proteins or transmembrane protein of influenza viruses. Among these, M2e is highly conserved across different influenza virus subtypes, while HA and NA are the primary targets in research focused on broad-spectrum neutralizing antibodies [31,32]. All three proteins represent promising candidates for the development of a universal influenza vaccine. We used various immunoinformatics tools to select conserved polypeptide sequences with multiple dominant epitopes for a novel candidate of a universal influenza vaccine. To increase the potential protection effect, each polypeptide was conjugated to the immunostimulatory peptide Ii-Key to increase its immunogenicity [33], and the use of MF59 adjuvant was incorporated to enhance the immunogenic response [34,35].

The combination of polypeptides must meet the essential criteria of antigenicity and immunogenicity, which are fundamental characteristics required for vaccine candidates [36,37]. Our results indicate that the five selected polypeptides presented various degrees of binding affinity with specific IgG antibodies against different influenza virus subtypes. This initial screening allowed us to identify three promising polypeptide combinations for the evaluation of immunogenicity in mice. The results suggest that higher-dose polypeptides induced higher antibody levels, and P125-H induced neutralizing antibodies against various influenza viruses and the highest titers of average IgG antibodies against itself as well as significantly higher HI antibody titers against various influenza viruses compared to the other two polypeptide combinations. The results show a good consistence with the prediction scores of both MHC class II binding affinity and CD4 T-cell epitope recognition, showing that polypeptides with higher scores induced higher antibody levels. Our results show that P125-H induced the production of HI and neutralizing antibodies against H1N1 and H5N1 as well as H3N2 and H7N9, indicating that it induced both influenza Group 1 (H1, H5) and Group 2 (H3, H7) HA antibodies and has cross-protection capability [38,39]. In addition, P125-H immunization induced a high level of cell immune response, as shown by the high level of IFN-γ production in the splenic lymphocytes of the immunized mice under the stimulation of P125. These results demonstrate that the selected polypeptides have a potential to become a candidate for a universal influenza vaccine to provide broad-spectrum protection across different influenza subtypes.

P125-H provided an efficient protection on mice with a lethal infection of H1N1 or H7N9 virus. The results of viral challenge show P125-H significantly decreased weight loss and presented earlier weight recovery in mice with H1N1-CA04 or H7N9-MA, and reduced mortality by 30% and 50% in mice challenged with H1N1-CA04 and H7N9-MA, respectively. Compared to inactivated or live-attenuated vaccines targeting a single subtype, the protective efficacy of our vaccine may not be ideal. However, it offered a cross-protection against different subtypes, and the immunogenic components can be replaced at any time, affording it the potential to become a broad-spectrum vaccine. Both histopathological analysis and LB ratio data also suggested that P125-H reduced the lung injury of mice with H1N1-CA04 or H7N9-MA infection. Meanwhile, viral titration and IHC results suggest that P125-H decreased the viral load or localized viral distribution in the lungs of mice. In addition, compared to the adjuvant group, the levels of IFN-γ in the sera of immunized mice infected with H1N1-CA04 or H7N9-MA were significantly elevated. IFN-γ, a key cytokine secreted by Th1 cells, plays a critical role in cell-mediated immunity, and its production is beneficial for protective immunity against viral infections [40,41]. Collectively, the results from in vitro neutralization assays and viral challenge experiments suggest that the P125-H possesses a degree of broad-spectrum protective potential.

Antigenic drift and antigenic shift in influenza viruses present formidable obstacles to universal vaccine development. Whereas P125-H targets highly conserved epitopes, it would be easy to mitigate these challenges. Compared to vaccines using a single protein component, such as M2 or HA [42,43], our design incorporates a novel approach by employing the tandem combination of different proteins for immunization. This strategy effectively integrates antigenic epitopes from multiple viral proteins, thereby covering a broader range of virus subtypes and variants. Our research suggests that a strategy combining immunogenic polypeptide fragments from conserved regions of various influenza virus subtypes and proteins may offer a viable approach for designing a universal influenza vaccine [44]. However, there is still much research and design needed to transition from broad-spectrum vaccines to truly universal influenza vaccines. It would also be important to assess the basic toxicity as well as the protective efficacy of the combined polypeptides in different scenarios, such as models of previous infection, aged or juvenile subjects, pregnant women, or immunocompromised populations.

## 5. Conclusions

Our study highlights the development and evaluation of P125-H, a novel polypeptide-based universal influenza vaccine candidate. Leveraging immunoinformatics, conserved epitopes from multiple influenza virus subtypes were identified and fused using Ii-Key technology to enhance T-helper-cell responses. The vaccine demonstrated broad-spectrum immunogenicity, eliciting cross-reactive antibodies and robust cellular immune responses. Moreover, it provided significant protection against lethal influenza challenges in a mouse model by reducing viral loads and lung damage. These findings underscore the potential of P125-H as a universal influenza vaccine. Further studies are warranted to validate its efficacy across diverse populations and influenza virus strains.

## Figures and Tables

**Table 1:** Polypeptide epitope prediction tools, websites, and selection scores. * Note: In the NetMHCIIpan 4.1 EL prediction, the rank value represents the potential binding affinity of antigenic peptide fragments with MHC molecules. A rank < 10 indicates that this peptide’s binding affinity is in the top 10% of predicted peptides, meaning it binds more strongly than 90% of other peptides and has a higher immunogenicity. The IEDB CD4+ T-cell immunogenicity tool predicts parts of peptides that can bind to MHC II molecules. The higher the immunogenicity score, the stronger the binding potential. To select better peptides, we set the threshold at 90 (the highest threshold recommended by the website). VaxiJen v2.0 is used to predict protective antigens, with a default threshold of 0.4, meaning only peptides with a score greater than 0.4 are considered to be protective antigens. Bepipred Linear Epitope Prediction 2.0 predicts linear B-cell epitopes, with a threshold of 0.5 where sensitivity and specificity are balanced, facilitating a more comprehensive selection of high-quality peptides.

Predicted Structure	Tool/Algorithm	Website	Setting *
MHC-II binding	NetMHCIIpan4.1 EL	http://tools.iedb.org/mhcii/ (accessed on 18 August 2023)	Percentile Rank < 10
CD4+ T-cell immunogenicity	IEDB recommended (combined)	http://tools.iedb.org/CD4episcore/ (accessed on 24 August 2023)	Score Threshold > 90
Protective antigens	VaxiJen v2.0	https://www.ddg-pharmfac.net/vaxijen/VaxiJen/VaxiJen.html (accessed on 3 September 2023)	Threshold = 0.4
Linear B-cell epitopes	Bepipred Linear Epitope Prediction 2.0	http://tools.iedb.org/bcell/ (accessed on 12 September 2023)	Threshold = 0.5

## Data Availability

The original contributions presented in the study are included in the article. Further inquiries can be directed at the corresponding author.
